# Perinatal depression, perinatal mental health, and legal interventions: a medico-legal anthropological concept

**DOI:** 10.1007/s44192-025-00204-7

**Published:** 2025-05-15

**Authors:** Ritika Behl

**Affiliations:** 1 Alliance School of Law, Alliance University, Bengaluru, India; 2 Faculty of Law, Symbiosis International (Deemed University), Pune, India

**Keywords:** Perinatal depression, Perinatal mental health, Medico-legal, Anthropology, Psychiatry, Health policies

## Abstract

Perinatal depression (PND), and perinatal mental health (PMH) are regarded as an underestimated public health concern. A doctoral study was undertaken to analyze the efficacy of existing health laws and policies in India in addressing and managing PND, and the implications of non-recognition of PND as a public health issue. The interdisciplinary and transdisciplinary study involved four disciplines: Psychiatry, Medical Anthropology, Public Health Law, and International Human Rights Law. The study highlighted the need for synergistic assimilation of knowledge from the four disciplines to read mental health, PND, and PMH. The study novelly emphasized the need to read PND as a ‘medico-legal anthropological’ concept, which manifests medically, results largely, and is strongly influenced by psychosocial factors, making it imperative to recognize perinatal women as a vulnerable population and safeguard their human rights. This approach will facilitate assessments about interventions for addressing and/or managing PND when policy-making/policy reforms regarding PND are in progress.

## Introduction

Perinatal depression (PND) and perinatal mental health (PMH) are regarded as underestimated public health issues, regardless of their direct bearing on women’s rights [1, 2]. The policy gap concerning addressal, and management of PND in India has been underscored recently [3–5]. Also, the implications of deprioritization of PMH and non-management of PND have been recognized as a precursor of human rights violations, especially women’s rights [2, 3].

## PND’s disease burden and new motherhood: care labour by Impaired Perinatal Women in Patriarchal Indian Society

Depression, a mental disorder, results in impaired quality of life, social functioning, and workplace participation besides widely affecting the caregivers of the affected person and communities at large [6, 7]. The first thousand days of a child’s life, from the antenatal period to the first two years are immensely critical for their physical development [8]. The infants depend on their caregivers for their physical, cognitive, and social development which is affected in multidimensional ways by the caregiver’s mental health.

Women are expected to be the primary caregiver for the infant [9–11], even in times of impaired functionality attributable to PND [12]. Besides that, they are expected to fulfill the surmounting expectations of families, and society, where they ought to perform their duties as a daughter-in-law, (happy) new mother, and a wife, in addition to taking responsibility for household chores [13, 14]. These cultural expectations created over the years are strongly supported by patriarchal views which are ingrained in Indian society [13, 14]. More so, perinatal women also, instead of self-empathizing, start criticizing themselves if they are unable to meet these enormous expectations [15].

In the absence of guidelines or protocols to ensure screening, detection, or diagnosis of perinatal mental disorders (PMDs) including PND, perinatal women continue to remain unaware of their deteriorated PMH condition nor are their partner or family members made aware of the same [16, 17]. In India, where most caregivers and families are reluctant to seek out professional support for mental health issues, appreciating and understanding the need for a mental health support system for perinatal women can play an extraordinary role [18].

More so, social determinants of health potentially affect women’s health and their caregiving ability for their newborns and their other children, besides influencing their roles as consumers and providers of healthcare for their infants and/or other children [19–21]. More so, since PMH issues are accepted to be part and parcel of pregnancy and new motherhood, women are generally left alone to deal with them [13].

The Indian Psychiatric Society’s presidents have played a vital role in furthering the importance of women’s mental health. The former president Dr. E. Mohan Das created the ‘Task Force of Women Mental Health’ in the year 2009 which was responsible for underscoring an update on women’s mental health in special relation to Indian society [13]. The task force was supposed to formulate guidelines for the pharmacological treatment of mental disorders in women occurring during the antenatal and postnatal period and to undertake activities on different aspects of women’s mental health [13].

Dr. Ajit Avasthi furthered the ongoing work and created the ‘Committee on Women’s Mental Health’ [13]. In 2011, the practice guidelines for the treatment of women with psychiatric disorders during pregnancy and lactation period were published for Indian psychiatrists [13]. Later it was proposed that guidelines for married women with mental illness should be released but the project received objections because of the involvement of sensitive issues like highlighting the gender-based roles of women as mothers and primary care providers for children, glorification of motherhood, and unrealistic expectations from new mothers, etc. [13].

Thus, the progression of research on women’s mental health in India has been affected by various roadblocks where it has not been given the required attention and resources. More so, due to the small number of female psychiatrists in India (14.6%) especially in senior positions (10%), women’s mental health has been severely overlooked [22].

## Mental health, PND, and PMH: need for synergistic assimilation of knowledge from the four disciplines: Psychiatry, Medical Anthropology, Public Health Law, and International Human Rights Law

No group or institution can pursue the healthcare justice movement in isolation. There must be a coalition and amalgamation of joint efforts by different stakeholders including individuals, non-state actors, health personnel, and governments. Such efforts must be intranational and international to achieve the highest attainable standard of health globally. It must be understood as a chain of events or movements that will have a ripple effect and such impact must be molded according to the needs of the communities. Also, mere provisions of human rights are not sufficient. They must be claimed actively, and fulfill the needs of the population.

Gostin notes that fulfilling the rights will cast positive obligations on the States and entail legislative, financial, and promotional activities, where the right-holders will acquire the capacity to claim and assert such rights for themselves and others [23]. Krennrich also observes that for human rights to have the necessary effect they need to be claimed, which makes human rights empowerment essential [24].

Public health largely denotes the State’s performance in concretizing the ‘right to health’, and is viewed as a State’s obligation under International Human Rights Law. Also, under the ‘right to health’, the State must ensure the provision of facilities, goods, services, and conditions necessary for the attainment and maintenance of the highest attainable standards of health [25]. It is also imperative to note here that the efficacy of the Public Health Law is intertwined with the protection of human rights, where its disregard or ignorance can risk making individuals marginalized [26]. Consequently, for these marginalized people, the right to health will suffer, and the goal of the highest attainable standard of health will stand vitiated [26].

Also, the right to health implies a right to participate in the decision-making process affecting health, and, therefore, can be linked with “active social citizenship” which further broadens the concept of health, and includes social determinants of health [27]. More so, it can be stated that the right to health has “two basic components: a right to health care, and a right to healthy conditions” [27]. Hence, a holistic approach to health becomes incumbent and encompasses healthcare and social conditions [27]. Also, public health doctrine involves enhanced awareness, and recognition of the role of social determinants of health, making it indispensable to address health inequities through population-based policies that can equitably facilitate health improvement [27].

Therefore, Medical Anthropology also plays an inherent role in protecting and enjoying the right to health. Hence, adopting a rights-based approach to the right to health and public health is deeply influenced by the interplay between Public Health Law and Medical Anthropology. To improve population health through population-level interventions, a critical role is played by medical care in preventing mortality, and in improving quality of life, due to which clinical medicine, and public health converge in multiple ways.

Also, the human rights approach underscores the inherent integrity of individuals, their entitlements, and the corresponding State’s obligations, which the State freely undertakes by ratifying human rights instruments. Therefore, it becomes imperative for States to protect the entire population’s ‘right to health’ through legislative, and other measures. Consequently, State agencies, private-sector, and other non-state actors providing health services must ensure no discrimination in access to health facilities, and services, apart from their quality, and promotive education.

This would require the State to intervene when individuals cannot realize their ‘right to health’ because of factors beyond their control. Hence, it can be specified that the ‘right to public health’ is intrinsically linked to the ‘right to health’, and primarily deals with the legal relationship between a State, and individuals under its jurisdiction [27]. More so, since the ‘right to health’ provides for preventive, promotive, curative, and palliative healthcare [25], it further facilitates intersections between the four disciplines of International Human Rights Law, Public Health Law, Medical Anthropology, and Clinical Medicine (Psychiatry in the case of PND since it provides its nosology).

This approach can facilitate the achievement of population-based health targets through the principles of health equity and health promotion while elevating the health status of the marginalized obligatory through the theory of “right to health-based public health” [27].

## PND: a medico-legal anthropological concept

PND must be read from three different facets, including: -

1. as a mental disorder,
2. as a public health issue,
3. as a precursor of human rights violations (when it is (adequately) not addressed and/or managed owing to policy gaps).

Also, PND needs to be recognized as a Medico-Legal Anthropological concept:

- which manifests medically (screening, detection, and treatment are embedded in its psychiatric nosology (The American Psychological Association has defined nosology as “the scientific study and classification of diseases and disorders, both physical and mental” [28])),
- results largely, and are strongly influenced by the psychosocial factors that exist in society [10, 29] that consistently and continuously interact with each other [30, 31]; and whose impact is magnified in the absence of requisite health laws and policies [17], and
- making it imperative to recognize perinatal women as a vulnerable group of the population, as previously signified by the WHO [32], Committee on the Elimination of Discrimination Against Women [33], and Committee on the Rights of the Child [34], since their human rights are violated because of (mental) health inequity; which is attributable to biological/hormonal reasons, and/or psychosocial-cultural reasons; and the violations aggravate due to the pervading policy gaps or lapses/issues in implementation of health laws and policies.

Thereby, laws play a cardinal role in attaining the health goals of the population, and in protecting the vulnerable groups of the population, which warrants the introduction of targeted health laws and policies for PND, and amendment of existing laws to recognize PND as a public health issue [35].

- Also, when assessments about interventions to address and/or manage PND are being made, or when policy-making/policy reforms regarding PND are in progress.
- It is also essential to take note of those policy reforms or law reforms which have been reported to be effective and influential in meeting perinatal mental health needs of women in other different countries, especially those having similar psychosocial, and cultural environments like the South-East Asian Region, or those affected by economic resource constraints like Ethiopia.

Sri Lanka has made significant strides through their policy reforms and agendas in meeting women’s PMH needs and in reducing perinatal maternal mortality rates [36]. Likewise, in Thailand apart from providing PMH services at the primary care level, planning is underway to train the non-specialist as well [37]. In Ethiopia, the National Mental Health Strategy 2020–25 apart from the National Mental Health Strategy 2012-13–2015-16 has prioritized meeting maternal mental health needs and remedied the existing policy gaps [38].

This approach can be found effective in estimating not just the impact of law and policy reforms but, also, in helping understand the different interventions that have been prioritized by other countries relating to PMDs and PMH.

The following reasons justify the description of PND and PMH as a ‘medico-legal anthropological’ concept:

1. The understanding and literature about PND should not remain limited to the field of psychiatry or its psychiatric nosology as a mental disorder. The psychiatric nosology of PND remains crucial in defining its nature and its symptoms, and in attaining updated information about the treatments (health interventions) that can be offered.
2. It has been consistently reported that PND is triggered or exacerbated not only because of biomedical factors including biological, hormonal, or psychiatric factors but also because social determinants of health play a critical role [10, 29]. This aspect becomes even more significant considering that the factors associated with the etiology of PND are multitudinal and cyclical [30, 31].

It can be termed cyclical because PND which might onset because of social determinants and/or biological factors, can be worsened due to the sustained influence of social forces, and can also relapse, due to the same risk factors or resulting from a cumulative effect of other risk factors [30, 31]. Also, these factors comprising the etiology of PND tend to interact and affect, cumulatively, like a web [39, 40].

More so, cultural constructs of pregnancy, motherhood, and mental health largely affect service delivery and utilization for PND [10, 11], which warrants an understanding of its social perception in a given community. Such understanding will facilitate assessments about the need, scope, and type of health, and legal interventions that must be delivered for PND. Furthermore, since perinatally depressed women’s functioning tends to be impaired [31], they may not be in a position to safeguard themselves against the adversely influencing social determinants of health.

These reasons make it incumbent to contemplate that the nature of PND is ‘anthropological’ since multiple, and multifarious social forces play a cardinal role in ‘affecting’, and ‘protecting’ the mental health and well-being of perinatal women. Hence, the discipline of Medical Anthropology becomes intrinsically attached to the addressal, and management of PND.

3. Furthermore, the discipline of Public Health Law, and International Human Rights Law become inseparably attached to PND, and PMH due to the following facets:
  - Role of existing health laws and policies in recognition of PND as a public health issue, in delivering quality PMH services, and to assess if mental health needs of perinatal women are being met in consonance with the human rights treaty obligations undertaken by a State.
  - To assess whether the effect of social determinants associated with the onset or exacerbation of PND is mitigated through legal interventions.
  - The need to introduce or restructure existing health laws and policies arises from the above factors.

Also, in addressing and managing PND, none of the disciplines including Psychiatry, Medical Anthropology, Public Health Law, and International Human Rights Law should act as subordinate to each other but should inform each other. Consequently, the outcome must reflect the synergistic assimilation of knowledge from these disciplines to ensure that results are largely universally beneficial.

This can be ensured through various ways. There should be a special, interdisciplinary task force established by the Governments comprising clinical specialists like psychiatrists, and non-specialists like social psychologists, anthropologists, lawyers, policymakers, and researchers based on their experience, expertise, and research in PMDs and PMH. Academic events like interdisciplinary and transdisciplinary conferences, such as the recently held 10 th World Congress on Women’s Mental Health, provided an eloquent platform for scientists and researchers from various disciplines to promote the integration of knowledge and work on multisectoral collaborations.

Platforms like these can help in the preparation, and updating of protocols, guidelines, etc. in the delivery of PMH services and related interventions. Medical associations like the Indian Psychiatry Society can also prepare the guidelines which can be submitted to different stakeholders like the Ministry of Health and Family Welfare and Ministry of Women and Child Development.

However, in this interplay, it is purported that ‘law’ must play an integral role in the following ways:

- In ensuring the availability of mental health treatments, and regulating the use of certain treatments (e.g. the District Mental Health Progarmme [DMHP]) [41] in India ensures the availability of mental health treatments including antidepressants at the district level, and under Sec. 94(3) of the Indian Mental Health Care Act, 2017 use of electroconvulsive therapy for treating mental health disorders has been prohibited.
- In altering the impact of social determinants through the existing health laws, and policies (e.g. introduction of laws against female infanticide, and female feticide for combating gender bias against the girl child in Indian society).

Behl and Nemane [42] discussed how the Indian criminal justice dispensation is deeply impacted by the policy gaps relating to PMDs and PMH, where PMDs are not under-diagnosed or not diagnosed and no treatment is provided even to high-risk perinatal women. If women from these high-risk population groups commit infanticide, they have to face triple jeopardy of law: unprovided treatment services for a public health issue especially which is gender-related, being a perinatal woman offender who is tried in courts at par with non-perinatal population groups including men, and instead of being provided treatment and rehabilitation is incarcerated and punished.

- Introduction of legal interventions to protect or/and promote the rights of perinatal women, whose impact in terms of human rights may or may not be apparent (e.g. introduction of cash transfer schemes in India under Janani Suraksha Yojna [43] not only resulted in rise in institutional births but also resulted in decreasing maternal mortality, which in turn resulted in safeguarding ‘right to life’; and effective implementation of the Maternity Benefits Act, 1961 acts as a protective factor against PND).

4. Further, in the above-stated backdrop, the Public Health Law, and International Human Rights Law, conjointly play a cardinal role, keeping into consideration that the consequences of PND are not limited to the mother only but extend to her child [44], and family [45], and also impact society at large [46]. Thereby, PND should not be underscored as a ‘women-centric’ public health issue but must be recognized as a population-level public health concern. Hence, it must be realized that PND not only results in violation of women’s rights but also has far-fetched effects on children’s rights, and family rights, besides other human rights.

In this reference, the nexus between the ‘right to health’, and public health, in the context of International Human Rights Law, becomes significant because the latter provides for the normative and operational system. Under the normative theory of human rights, the ‘right to health’ provides for certain freedoms, and entitlements for individuals while, correspondingly, specific duties are imposed upon States to ensure the protection, and realization of the right and other correlated human rights [27].

The operating system provides for the enforcement of these rights, monitoring their enforcement, what amounts to a violation of these rights, who should be held accountable for it, and how [27]. Also, the “system theory” of International Law facilitates the functioning of normative and operational systems of International Human Rights Law [27].

India is a signatory to the core Human Rights Treaties including the International Covenant on Economic, Social and Cultural Rights (ICESCR), the Convention on the Elimination of All Forms of Discrimination Against Women (CEDAW), the Covenant on the Rights of the Child (CRC), Covenant on the Rights of Persons with Disabilities (CRPD). Non-addressal and non-management of PMDs and ensuring availability of adequate quality PMH services for women amounts to violations of treaty obligations under these core Human Rights Treaties. The detailed description of the International Human Rights Treaty obligations of signatory States related to PMDs and delivery of PMH services have been described elsewhere [47].

5. Moreover, ‘law’ becomes essential as a ‘tool’ in the form of legal interventions (laws and policies), and as a ‘medium’ for introducing/restructuring legal interventions, in the future, based on sustained synergistic assimilation of knowledge from other co-disciplines. For example, for recommending and banning treatments for managing PND at different levels in society, constant updating of health personnel and policymakers apart from law enforcement agencies is required.

Besides the above-stated, the legal interventions are also important from the following perspectives:

A. To promote protective health behavior at individual, community, and population levels by introducing health laws and policies providing PMH services.
B. To sensitize the law enforcement agencies including judges, lawyers/advocates, and police force about the nature and consequences of PND since the interface impacts legal systems in various dimensions. For example, cases involving matrimonial issues and divorce, attempted or completed neonaticide or infanticide, seeking permission for medical termination of pregnancy, maternity leave laws, etc.
C. Most importantly, for providing a framework for ensuring availability, accessibility, acceptability, and quality (AAAQ) of PMH services, and to underscore entities responsible for the same.

Also, ensuring that health laws and policies recognize PMDs including PND as a public health issue can act as a protective shield against the following factors:

- Molding of political will to deprioritize mental health especially women’s perinatal health, more so in the background of gender-based roles, and perception about motherhood in a patriarchal society like India.
- Allocation of financial resources for mental health remains a universal challenge. However, the implementation of such health laws and policies will facilitate the allocation of resources for PMH services or at least promote speedier integration of PMH services at the primary care level.
- Generate more community awareness about the importance of PMH and the treatability of PMDs in the backdrop of various misconceptions and pervasive knowledge gaps about PMDs amongst healthcare providers and utilizers, which should result in more demand and supply of PMH services.

## Conclusion

Significant progress can be achieved through multilevel and multisectoral collaborations between different ministries like the Ministry of Family and Health Welfare, Ministry of Women and Child Development, Ministry of Youth Affairs, Ministry of Social Justice and Empowerment, etc. Also, non-state actors following models of Postpartum Support International, Policy Center for Maternal Mental Health, etc must be promoted in the Indian setting to meet women’s PMH needs through community awareness programmes, tracking of progress made through policy initiatives or lacunas created owing to policy gaps, etc. Consistently highlighting India’s Human Rights Treaty obligations in this context by policymakers and non-state actors should facilitate the prioritization of PMH services within reproductive and mental health and rights frameworks.

In the present SDG era, it is imperative to recognize the increased vulnerability of perinatal women to PND while providing care labor for their newborns and other children while dealing with PMDs including PND in isolation. The present situation warrants recognizing PND as a public health issue and ensuring multisectoral efforts. Legal interventions and health intervention, concomitantly, can ensure that the disease burden arising from PND is managed which requires synergistic assimilation of knowledge, and non-subordination of any discipline over the other. Hence, a transdisciplinary and interdisciplinary approach must be adopted in assessing the interventions that must be delivered to meet the mental health needs of perinatal women.

## Data Availability

No datasets were generated or analysed during the current study.

## References

[1] Howard LM, Khalifeh H. Perinatal mental health: a review of progress and challenges. World Psychiatry. 2020;19(3):313–27. 10.1002/wps.20769. 32931106 10.1002/wps.20769 PMC7491613

[2] Behl R. Judicial interface with perinatal depression in India: an empirical analysis and thematic review of published judgments. Arch Womens Ment Health. 2023. 10.1007/s00737-023-01391-4. 37987837 10.1007/s00737-023-01391-4

[3] Behl R, Nemane V, Sims D. Perinatal mental disorders: the ‘Non Liquet’ facet of mental health legislative instruments in India. J Health Manage. 2024. 10.1177/09720634241236834.

[4] Kalra H, Thach TD, Loreno R, Sagar R, Fisher J. National policies and programs for perinatal mental health in India: A systematic review. Asian J Psychiatr. 2023;91: 103836. 37988929 10.1016/j.ajp.2023.103836

[5] Priyadarshini U, Rao AP, Dash S. Recommendations for maternal mental health policy in India. J Public Health Policy. 2023;44(1):90–101. 10.1057/s41271-022-00384-4. 36624268 10.1057/s41271-022-00384-4 PMC9827439

[6] Whiteford HA, Ferrari AJ, Degenhardt L, Feigin V, Vos T. The global burden of mental, neurological and substance use disorders: an analysis from the Global Burden of Disease Study 2010. PLoS ONE. 2015;10(2): e0116820. 10.1371/journal.pone.0116820. 25658103 10.1371/journal.pone.0116820 PMC4320057

[7] WHO, Mental disorders, 2022. https://www.who.int/news-room/fact-sheets/detail/mental-disorders.

[8] Black MM, Hurley KM. Investment in early childhood development. Lancet. 2014;384(9950):1244–5. 10.1016/S0140-6736(14)60607-3. 24947105 10.1016/S0140-6736(14)60607-3

[9] Shanbhag V, Chandra P, Desai G, Bagadia A, Le Dref M, Bhat S. If they don’t ask, we don’t share—a qualitative study on barriers and facilitators to discussing mental health with obstetric care providers in urban anganwadis among pregnant women in India. Indian J Social Psychiatry. 2023;39(3):215–20. 10.4103/ijsp.ijsp_117_23.

[10] Fellmeth G, Kanwar P, Sharma D, Chawla K, DasGupta N, Chhajed S, Chandrakant JEC, Thakur A, Gupta V, Bharti OK, Singh S, Desai G, Thippeswamy H, Kurinczuk JJ, Chandra P, Nair M, Verma A, Kishore MT, Alderdice F. Women’s awareness of perinatal mental health conditions and the acceptability of being asked about mental health in two regions in India: a qualitative study. BMC Psychiatry. 2023;23(1):829. 10.1186/s12888-023-05323-5. 37957589 10.1186/s12888-023-05323-5 PMC10644637

[11] Insan N, Weke A, Forrest S, Rankin J. Social determinants of antenatal depression and anxiety among women in South Asia: a systematic review and meta-analysis. PLoS ONE. 2022;17(2): e0263760. 10.1371/journal.pone.0263760. 35139136 10.1371/journal.pone.0263760 PMC8827460

[12] Srisurapanont M, Oon-Arom A, Suradom C, Luewan S, Kawilapat S. Convergent validity of the Edinburgh postnatal depression scale and the patient health questionnaire (PHQ-9) in pregnant and postpartum women: their construct correlations with functional disability. Healthcare (Basel). 2023;11(5):699. 10.3390/healthcare11050699. 36900704 10.3390/healthcare11050699 PMC10000426

[13] Sharma I, Pathak A. Women mental health in India. Indian J Psychiatry. 2015;57(Suppl 2):S201–4. 10.4103/0019-5545.161478. 26330635 10.4103/0019-5545.161478 PMC4539862

[14] Malhotra S, Shah R. Women and mental health in India: an overview. Indian J Psychiatry. 2015;57(Suppl 2):S205–11. 10.4103/0019-5545.161479. 26330636 10.4103/0019-5545.161479 PMC4539863

[15] Maitra S, Brault MA, Schensul SL, Schensul JJ, Nastasi BK, Verma RK, Burleson JA. An approach to mental health in low and middle income countries: a case example from urban India. Int J Ment Health. 2015;44(3):215–30. 10.1080/00207411.2015.1035081. 26834278 10.1080/00207411.2015.1035081 PMC4731031

[16] Ransing R, Kukreti P, Deshpande S, Godake S, Neelam N, Raghuveer P, Mahadevaiah M, Kataria D, Patil S, Puri M, Padma K. Perinatal depression-knowledge gap among service providers and service utilizers in India. Asian J Psychiatry. 2020;47: 101822. 10.1016/j.ajp.2019.10.002. 10.1016/j.ajp.2019.10.002 PMC10349034 31710947

[17] Behl R, Nemane V. Perinatal mental health and the justice delivery system in India. Med Leg J. 2023. 10.1177/00258172231180094. 37318067 10.1177/00258172231180094

[18] Kukreti P, Ransing R, Raghuveer P, Mahdevaiah M, Deshpande SN, Kataria D, Puri M, Vallamkonda OR, Rana S, Pemde HK, Yadav R, Nain S, Prasad S, Garg B. Stepped care model for developing pathways of screening, referral, and brief intervention for depression in pregnancy: a mixed-method study from development phase. Indian J Soc Psychiatry. 2022;38(1):12–20. 35418726 PMC9005084

[19] Raman S, Srinivasan K, Kurpad A, Razee H, Ritchie J. “Nothing special, everything is maamuli”: socio-cultural and family practices influencing the perinatal period in urban India. PLoS ONE. 2014;9(11): e111900. 10.1371/journal.pone.0111900. 25369447 10.1371/journal.pone.0111900 PMC4219795

[20] Gailits N, Mathias K, Nouvet E, Pillai P, Schwartz L. Women’s freedom of movement and participation in psychosocial support groups: qualitative study in northern India. BMC Public Health. 2019;19(1):725. 10.1186/s12889-019-7019-3. 31182064 10.1186/s12889-019-7019-3 PMC6558745

[21] Langer A, Meleis A, Knaul FM, Atun R, Aran M, Arreola-Ornelas H, Bhutta ZA, Binagwaho A, Bonita R, Caglia JM, Claeson M, Davies J, Donnay FA, Gausman JM, Glickman C, Kearns AD, Kendall T, Lozano R, Seboni N, Sen G, Sindhu S, Temin M, Frenk J. Women and Health: the key for sustainable development. Lancet. 2015;386(9999):1165–210. 10.1016/S0140-6736(15)60497-4. 26051370 10.1016/S0140-6736(15)60497-4

[22] Sood M, Chadda RK. Women psychiatrists in India: a reflection of their contributions. Indian J Psychiatry. 2010;52(Suppl 1):S396-401. 10.4103/0019-5545.69277. 21836713 10.4103/0019-5545.69277 PMC3146198

[23] Gostin LO. The human right to health: a right to the “highest attainable standard of health.” Hastings Cent Rep. 2001;31(2):29–30. 11478106

[24] Krennerich M. The human right to health. Fundamentals of a complex right. In: Klotz S, Bielefeldt H, Schmidhuber M, Frewer A, editors. Healthcare as a human rights issue: normative profile, conflicts and implementation. Bielefeld: Transcript Verlag; 2017. p. 23–54.

[25] Committee on Economic, Social and Cultural Rights, General Comment No. 14 on the Nature of State Parties‘ Obligations, 47, U.N. Doc. E/C.12/2000/4, 2000, https://docstore.ohchr.org/SelfServices/FilesHandler.ashx?enc=4slQ6QSmlBEDzFEovLCuW1AVC1NkPsgUedPlF1vfPMJ2c7ey6PAz2qaojTzDJmC0y%2B9t%2BsAtGDNzdEqA6SuP2r0w%2F6sVBGTpvTSCbiOr4XVFTq hQY65auTFbQRPWNDxL

[26] World Health Organization (WHO). Advancing the right to health: the vital role of law, 2017. https://apps.who.int/iris/bitstream/handle/10665/252815/9789241511384-eng.pdf?sequence=1&isAllowed=y.

[27] Lee P. The nexus between right to health and public health in the context of International Human Rights Law (L.L.M. Dissertation, National Taiwan University), 2013. 10.13140/2.1.5162.7204

[28] American Psychological Association [Internet]. Nosology [last updated April 19, 2018]. https://dictionary.apa.org/nosology

[29] Shorey S, Ng ED. Evaluation of a technology-based peer-support intervention program for preventing postnatal depression (part 2): qualitative study. J Med Internet Res. 2019;21(8): e12915. 10.2196/12915. 31469080 10.2196/12915 PMC6740164

[30] Tol WA, Murray SM, Lund C, Bolton P, Murray LK, Davies T, Haushofer J, Orkin K, Witte M, Salama L, Patel V, Thornicroft G, Bass JK. Can mental health treatments help prevent or reduce intimate partner violence in low- and middle-income countries? A systematic review. BMC Womens Health. 2019;19(1):34. 10.1186/s12905-019-0728-z. 30764813 10.1186/s12905-019-0728-z PMC6376658

[31] Gupta S, Kishore J, Mala YM, Ramji S, Aggarwal R. Postpartum depression in north Indian women: prevalence and risk factors. J Obstet Gynaecol India. 2013;63(4):223–9. 10.1007/s13224-013-0399-x. 24431646 10.1007/s13224-013-0399-x PMC3763048

[32] World Health Organization (WHO). Guide for integration of perinatal mental health in maternal and child health services, 2022. https://www.who.int/publications/i/item/9789240057142.

[33] Committee on CEDAW. General Recommendation No. 18: Disabled Women. U.N. Doc. A/46/38, 1993. https://www.legal-tools.org/doc/ba95ff/pdf/

[34] Committee on the Rights of the Child. CRC General Comment No. 4: Adolescent Health and Development in the Context of the Convention on the Rights of the Child. U.N. Doc. CRC/GC/2003/4, 2003. https://www.ohchr.org/sites/default/files/Documents/Issues/Women/WRGS/Health/GC4.pdf

[35] Wang G. The impact of social and economic indicators on maternal and child health. Soc Indic Res. 2014;116:935–57.

[36] Palfreyman A. Addressing psychosocial vulnerabilities through antenatal care-depression, suicidal ideation, and behavior: a study among urban Sri Lankan women. Front Psychiatry. 2021;12: 554808. 10.3389/fpsyt.2021.554808. 34108890 10.3389/fpsyt.2021.554808 PMC8180592

[37] Abdelghaffar W, Daoud M, Philip S, Ransing R, Jamir L, Jatchavala C, Pinto da Costa M. Perinatal mental health programs in low and middle-income countries: India Thailand and Tunisia. Asian J Psychiatry. 2023;88: 103706. 10.1016/j.ajp.2023.103706. 10.1016/j.ajp.2023.103706 37536081

[38] Ministry of Health, Federal Democratic Republic of Ethiopia [Internet]. The National Mental Health Strategy 2020 2025 (cited Dec. 7, 2016). http://repository.iifphc.org/bitstream/handle/123456789/1423/MENTALHEALTH%20strategy.pdf?sequence=1&isAllowed=y

[39] Dimcea DA, Petca RC, Dumitrașcu MC, Șandru F, Mehedințu C, Petca A. Postpartum depression: etiology, treatment, and consequences for maternal care. Diagnostics (Basel). 2024;14(9):865. 10.3390/diagnostics14090865. 38732283 10.3390/diagnostics14090865 PMC11083152

[40] Yang K, Wu J, Chen X. Risk factors of perinatal depression in women: a systematic review and meta-analysis. BMC Psychiatry 2022;22(1):63. 10.1186/s12888-021-03684-3. 10.1186/s12888-021-03684-3 PMC8793194 35086502

[41] Ranade K, Kapoor A, Fernandes TN. Mental health law, policy & program in India—a fragmented narrative of change, contradictions and possibilities. SSM—Mental Health, 2022.

[42] Behl R, Nemane V. Perinatal mental health and the justice delivery system in India. Med Leg J. 2025;93:78–81. 10.1177/00258172231180094. 37318067 10.1177/00258172231180094

[43] Press Release, Ministry of Health and Family Welfare. (Dec. 30, 2008, 12:56 PM). Ensuring Safe Motherhood Through JSY. https://pib.gov.in/newsite/erelcontent.aspx?relid=46232

[44] Wisner KL, Miller ES, Tandon D. Attention to prevention-can we stop perinatal depression before it starts? JAMA Psychiat. 2019;76(4):355–6. 10.1001/jamapsychiatry.2018.4085. 10.1001/jamapsychiatry.2018.4085 PMC6685198 30747943

[45] Moore Simas TA, Huang MY, Patton C, Reinhart M, Chawla AJ, Clemson C, Eldar-Lissai A. The humanistic burden of postpartum depression: a systematic literature review. Curr Med Res Opin. 2019;35(3):383–93. 10.1080/03007995.2018.1552039. 30468090 10.1080/03007995.2018.1552039

[46] Lund C, Breen A, Flisher AJ, Kakuma R, Corrigall J, Joska JA, Swartz L, Patel V. Poverty and common mental disorders in low and middle income countries: a systematic review. Soc Sci Med. 2010;71(3):517–28. 10.1016/j.socscimed.2010.04.027. 20621748 10.1016/j.socscimed.2010.04.027 PMC4991761

[47] Behl R. Perinatal mental disorders, mental health services, and human rights treaties: recognizing the state’s obligations. Arch Womens Ment Health (manuscript in peer review) 10.1007/s00737-025-01604-y 40580342

